# Efficacy and safety of vunakizumab in moderate-to-severe plaque psoriasis patients with different body mass index: a *post hoc* analysis based on a phase III trial

**DOI:** 10.3389/fphar.2025.1685072

**Published:** 2026-01-06

**Authors:** Xin Wang, Linfeng Li

**Affiliations:** Department of Dermatology, Beijing Friendship Hospital, Capital Medical University, Beijing, China

**Keywords:** vunakizumab, moderate-to-severe plaque psoriasis, body mass index, efficacy, safety

## Abstract

**Objective:**

Vunakizumab is effective and safe for treating moderate-to-severe plaque psoriasis patients. This *post hoc* analysis was intended to assess the effects of vunakizumab in patients with different body mass index (BMI).

**Methods:**

In the phase III trial of vunakizumab (NCT04839016), 461 moderate-to-severe plaque psoriasis patients receiving vunakizumab were enrolled and categorized into baseline BMI<24 kg/m^2^ (N = 179), 24≤BMI<28 kg/m^2^ (N = 183), and BMI≥28 kg/m^2^ (N = 99) groups. At least 75% improvement from baseline in the Psoriasis Area and Severity Index (PASI 75), PASI 90, PASI 100, static physician’s global assessment (sPGA) 0/1, patient-reported outcomes (PROs), serum concentration of vunakizumab, and adverse events from week 0 (W0)-W52 were recorded.

**Results:**

A lower BMI was associated with higher W0--W12 accumulating PASI 75, PASI 90, PASI 100, and sPGA 0/1 response rates. From W0--W52, a lower BMI was associated with higher PASI 75, PASI 90, and PASI 100 scores at most time points and was related to sPGA 0/1 response rates from W4--W48. With respect to PROs, higher BMI was related to increased mean dermatology life quality index scores at several time points but was not associated with the mean worst itch numerical rating scale, EuroQoL-5D (EQ-5D) utility index, EQ-5D visual analog scale score, or short form-36 mental/physical component score. A lower BMI was related to a higher mean serum concentration of vunakizumab. The incidences of any adverse events and most specific adverse events did not differ among the groups.

**Conclusion:**

A lower BMI is associated with a greater treatment response and quality of life in moderate-to-severe plaque psoriasis patients receiving vunakizumab.

## 1 Introduction

Psoriasis is a common, chronic, inflammatory dermatosis characterized by the formation of itchy, scaly, red patches on the skin (Bhagwat and Madke, 2023; Mrowietz et al., 2024). Psoriasis affects approximately 2.0%–3.0% of the global population, placing a considerable burden on public health (Sugumaran et al., 2024; Damiani et al., 2021). The goals for managing psoriasis are to relieve symptoms and increase quality of life, and the main treatment methods include phototherapy, topical therapies, systemic immune modulators, and biological agents (Lee and Kim, 2023; Ferrara et al., 2024). In recent years, with increasing understanding of psoriasis pathogenesis, biological agents that target interleukin (IL)-17, IL-23, and tumor necrosis factor-alpha have been widely applied for the treatment of psoriasis (Lee and Kim, 2023; Wride et al., 2024).

Vunakizumab is a newly developed IL-17A monoclonal antibody that is used to treat several autoimmune diseases (Keam, 2024). Previous studies have revealed the efficacy and safety profile of vunakizumab in patients with moderate-to-severe plaque psoriasis (Zhang et al., 2022; Yan et al., 2025). For example, a phase II trial revealed that vunakizumab increased the Psoriasis Area and Severity Index (PASI) and Physician’s Global Assessment (PGA) of 0/1 response rates, with good tolerability versus placebo in moderate-to-severe plaque psoriasis patients (Zhang et al., 2022). Another randomized, double-blind, placebo-controlled phase III trial (NCT04839016) reported that moderate-to-severe plaque psoriasis patients who received vunakizumab had an elevated treatment response and comparable adverse reactions to those who received placebo (Yan et al., 2025).

Notably, several baseline characteristics may influence the efficacy of IL-17 inhibitors in patients with psoriasis (Hjort et al., 2024; Loft et al., 2022; Egeberg et al., 2024; Anghel et al., 2021). Previous studies have revealed that treatment responses to several IL-17 inhibitors (such as secukinumab and brodalumab) could be affected by the body mass index (BMI) of patients with psoriasis (Huang et al., 2020; Rompoti et al., 2023). Currently, it remains unclear whether the treatment response to vunakizumab is distinct in moderate-to-severe plaque psoriasis patients with different BMIs.

Therefore, this *post hoc* analysis used data from a phase III trial (NCT04839016) aiming to investigate the efficacy and safety of vunakizumab in moderate-to-severe plaque psoriasis patients with different BMIs.

## 2 Methods

### 2.1 Study design and population

NCT04839016 is a double-blind, parallel, placebo-controlled, multicenter trial that randomly allocated 690 patients with moderate-to-severe plaque psoriasis to receive vunakizumab or placebo at a ratio of 2:1. Patients underwent 52 weeks of therapy (the entire period), which included a 12-week induction period followed by a 40-week maintenance period. Patients received 240 mg of vunakizumab or a matching placebo during the induction period, and all the patients continued or switched to vunakizumab during the maintenance period. The detailed procedures, randomizations, and interventions were published previously (Yan et al., 2025). Each center of the institutional review board approved this trial.

In the current *post hoc* study, patients in the vunakizumab group were selected (N = 461), and those in the placebo group were excluded.

### 2.2 Data collection and grouping

Analysis data were collected from NCT04839016, which included information on clinical characteristics, clinical responses, time to first achieve PASI 75/90 during the induction period, time to first achieve PASI 100 during the whole period, patient-reported outcomes (PROs), the serum concentration of vunakizumab, and adverse events. Patients were divided into the following groups according to their baseline body mass index (BMI) values: BMI <24 kg/m^2^, 24≤ BMI <28 kg/m^2^, and BMI ≥28 kg/m^2^. There were 179 patients in the BMI <24 kg/m^2^ group, 183 patients in the 24≤ BMI <28 kg/m^2^ group, and 99 patients in the BMI ≥28 kg/m^2^ group. The BMI thresholds used in our study (<24, 24–28, and ≥28 kg/m^2^) were based on the Chinese adult BMI classification recommended by the National Health Commission of China (WS/T 428–2013). According to these criteria, a BMI <24 kg/m^2^ was considered normal weight, 24–28 kg/m^2^ was considered overweight, and ≥28 kg/m^2^ was considered obese (available at https://www.chinesestandard.net/).

### 2.3 Assessment

In this *post hoc* study, the clinical responses, including the PASI 75, the PASI 90, the PASI 100, and the static physician’s global assessment (sPGA) 0/1, were used to assess the efficacy of vunakizumab. The efficacy endpoints were as follows: 1) accumulating clinical response rate from baseline to week 12 (W0--W12), which was defined as the proportion of patients who achieved a clinical response at least once during the induction period; 2) the clinical response rate over time through week 52; and 3) the probability of achieving a PASI score of 75/90 during the induction period and the probability of achieving a PASI score of 100 during the whole period. Moreover, PROs, which include the dermatology life quality index (DLQI) score (Finlay and Khan, 1994), worst itch numerical rating scale (WI-NRS) (Stander et al., 2022), EuroQoL-5D (EQ-5D) utility index, EQ-5D and visual analog scale (VAS), short form-36 mental component score (MCS) and physical component score (PCS) (Laucis et al., 2015), are used for health-related quality-of-life appraisal. In addition, adverse events were assessed for the safety of vunakizumab in treating psoriasis patients.

### 2.4 Statistics

SPSS 29.0 (IBM, United States) was used for this *post hoc* study, and *P* < 0.05 indicated statistical significance. Comparisons of continuous variables were performed via ANOVA. The comparison of classified variables among groups was completed via the *χ* ^2^ test or Fisher’s exact test. The median time with a 95% confidence interval (CI) of first achieving a PASI of 75/90 during the induction period and the median time with a 95% CI of first achieving a PASI of 100 during the whole period were calculated and are shown through Kaplan‒Meier curves. The median time to first achieve a PASI score of 75/90/100 among the different groups was compared via the log-rank test.

## 3 Results

### 3.1 Comparison of the clinical features of moderate-to-severe plaque psoriasis patients with different BMIs

A total of 461 moderate-to-severe plaque psoriasis patients, including 352 (76.4%) males and 109 (23.6%) females, had a mean age of 41.7 ± 13.2 years. There were 179 patients with a BMI <24 kg/m^2^, 183 patients with a 24≤ BMI <28 kg/m^2^, and 99 patients with a BMI ≥28 kg/m^2^. Age (*P* < 0.001), sex (*P* = 0.005), the incidence of hypertension (*P* = 0.002), and the prevalence of hyperuricemia (*P* = 0.008) differed among patients with different BMIs. No discrepancy was observed in other clinical features among patients with different BMIs (all *P* > 0.05). More information on moderate-to-severe plaque psoriasis patients is listed in Table 1.

**TABLE 1:** Clinical characteristics of PsO patients with different BMIs.

Characteristics	Total (N = 461)	BMI <24 kg/m^2^ (n = 179)	24≤ BMI <28 kg/m^2^ (n = 183)	BMI ≥28 kg/m^2^ (n = 99)	*P* value
Age (years), mean ± SD	41.7 ± 13.2	39.5 ± 14.2	44.7 ± 12.7	40.1 ± 11.2	<0.001
Sex, n (%)					0.005
Male	352 (76.4)	123 (68.7)	145 (79.2)	84 (84.8)	
Female	109 (23.6)	56 (31.3)	38 (20.8)	15 (15.2)	
Smoking, n (%)					0.092
Never	249 (54.0)	103 (57.5)	102 (55.7)	44 (44.4)	
Former or current	212 (46.0)	76 (42.5)	81 (44.3)	55 (55.6)	
Family history of PsO, n (%)					0.375
No	368 (79.8)	148 (82.7)	145 (79.2)	75 (75.8)	
Yes	93 (20.2)	31 (17.3)	38 (20.8)	24 (24.2)	
Hypertension, n (%)					0.002
No	387 (83.9)	163 (91.1)	142 (77.6)	82 (82.8)	
Yes	74 (16.1)	16 (8.9)	41 (22.4)	17 (17.2)	
Hyperlipemia, n (%)					0.240
No	386 (83.7)	155 (86.6)	153 (83.6)	78 (78.8)	
Yes	75 (16.3)	24 (13.4)	30 (16.4)	21 (21.2)	
DM, n (%)					0.927
No	439 (95.2)	170 (95.0)	174 (95.1)	95 (96.0)	
Yes	22 (4.8)	9 (5.0)	9 (4.9)	4 (4.0)	
Hyperuricemia, n (%)					0.008
No	408 (88.5)	164 (91.6)	165 (90.2)	79 (79.8)	
Yes	53 (11.5)	15 (8.4)	18 (9.8)	20 (20.2)	
Disease duration, n (%)					0.911
<2 years	67 (14.5)	27 (15.1)	25 (13.7)	15 (15.2)	
≥2 years	394 (85.5)	152 (84.9)	158 (86.3)	84 (84.8)	

PsO, psoriasis; BMI, body mass index; SD, standard deviation; DM, diabetes mellitus.

### 3.2 Comparison of treatment response in moderate-to-severe plaque psoriasis patients with different BMIs

**FIGURE 1:**
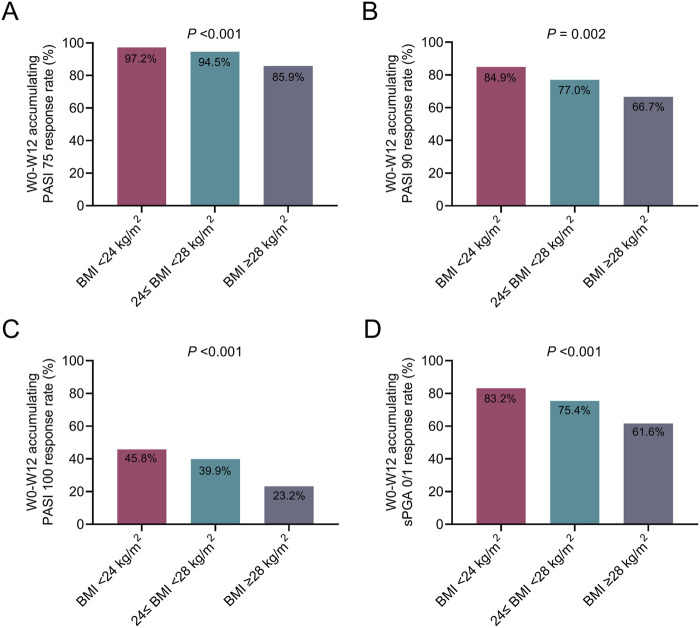
W0-W12 cumulative treatment response in moderate-to-severe plaque psoriasis patients with different BMIs. Comparison of the W0--W12 cumulative PASI 75 response rates **(A)**, PASI 90 response rates **(B)**, PASI 100 response rates **(C)**, and sPGA 0/1 response rates **(D)** among moderate-to-severe plaque psoriasis patients with BMI <24 kg/m^2^, 24≤ BMI <28 kg/m^2^, and BMI ≥28 kg/m^2^. Comparisons of these rates among the three groups were carried out via the χ^2^ test or Fisher’s exact test. P < 0.05 indicated statistical significance.

The W0--W12 accumulating PASI 75 response rates (97.2% vs. 94.5% vs. 85.9%, *P* < 0.001), PASI 90 response rates (84.9% vs. 77.0% vs. 66.7%, *P* = 0.002), PASI 100 response rates (45.8% vs. 39.9% vs. 23.2%, *P* < 0.001), and sPGA 0/1 response rates (83.2% vs. 75.4% vs. 61.6%, *P* < 0.001) were the highest in patients with BMI <24 kg/m^2^, followed by patients with 24≤ BMI <28 kg/m^2^, and the lowest in patients with BMI ≥28 kg/m^2^ (Figures 1A–D). According to the multivariable logistic regression analysis, it was shown that BMI ≥28 kg/m^2^ (vs. BMI <24 kg/m^2^) was independently associated with lower PASI 75/90/100 rates at W12 (all *P* < 0.05) (Supplementary Table S1).

**FIGURE 2:**
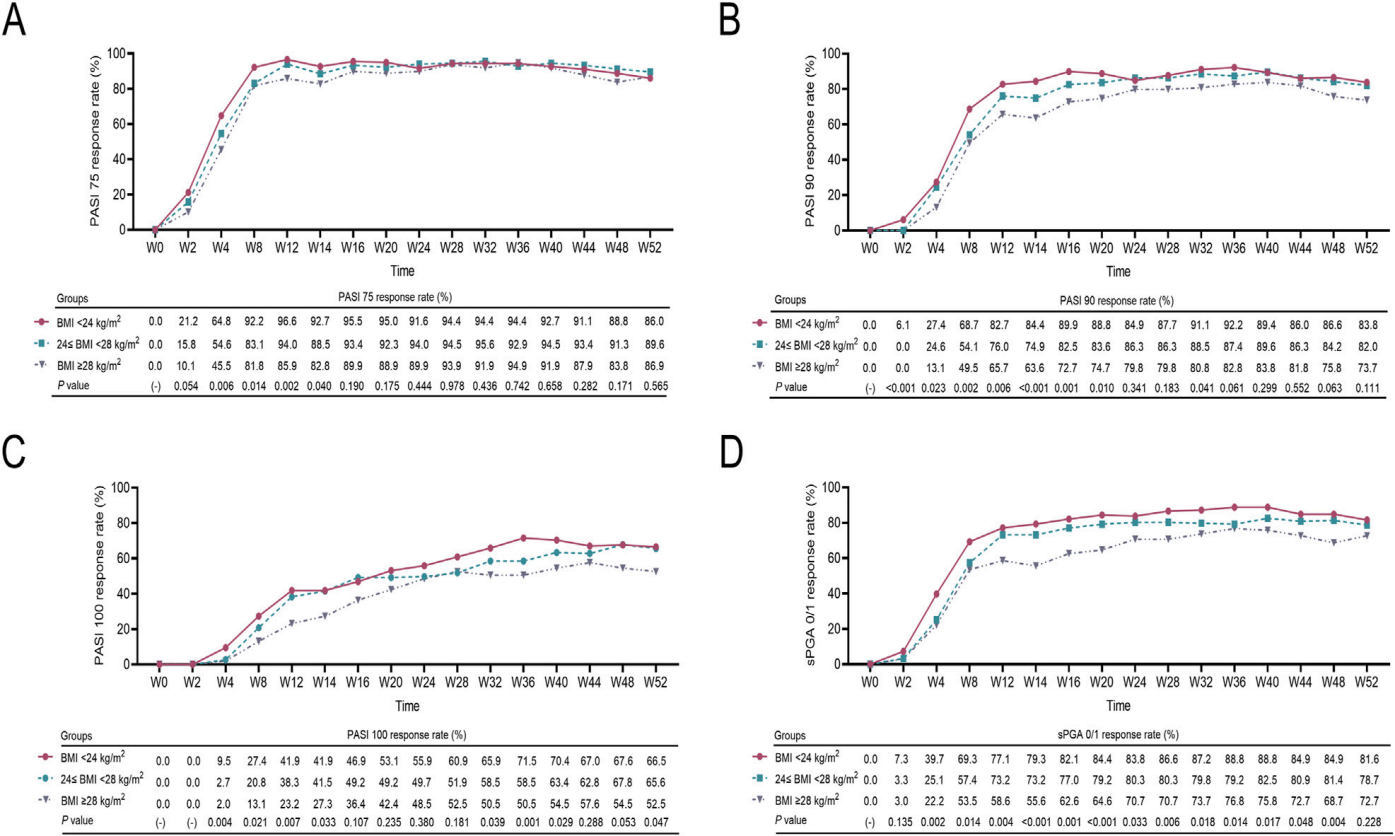
W0-W52 treatment response in moderate-to-severe plaque psoriasis patients with different BMIs. Comparison of W0-W52 PASI 75 response rates **(A)**, PASI 90 response rates **(B)**, PASI 100 response rates **(C)**, and sPGA 0/1 response rates **(D)** among moderate-to-severe plaque psoriasis patients with BMI <24 kg/m^2^, 24≤ BMI <28 kg/m^2^, and BMI ≥28 kg/m^2^. Comparisons of these variables among the three groups were carried out via ANOVA. *P* < 0.05 indicated statistical significance.

The PASI 75 response rate, PASI 90 response rate, PASI 100 response rate, and sPGA 0/1 response rate from W0 to W52 gradually increased first and then remained stable in patients with different BMIs. The PASI 75 response rate from W4 to W14, the PASI 90 response rate from W2 to W20 and W32, the PASI 100 response rate from W4 to W14, from W32 to W40, and W52, as well as the sPGA 0/1 response rate from W4 to W48, were the highest in patients with a BMI <24 kg/m^2^, moderate in patients with 24≤ BMI <28 kg/m^2^, and the lowest in patients with a BMI ≥28 kg/m^2^ (all *P* < 0.05) (Figures 2A–D).

### 3.3 Comparison of PROs in moderate-to-severe plaque psoriasis patients with different BMIs

**FIGURE 3:**
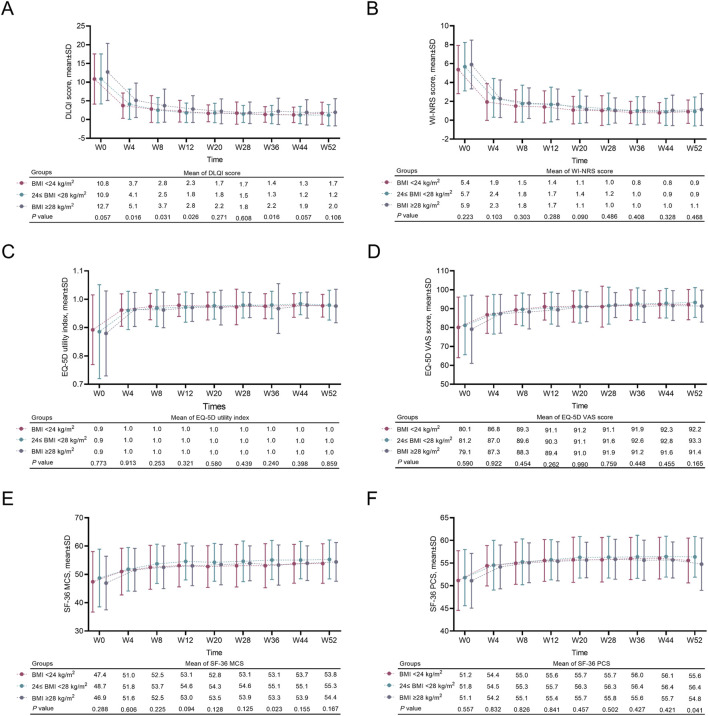
W0-W52 PRO in moderate-to-severe plaque psoriasis patients with different BMIs. Comparison of W0-W52 mean DLQI scores **(A)**, WI-NRS scores **(B)**, EQ-5D utility indices **(C)**, EQ-5D VAS scores **(D)**, SF-36 MCS scores **(E)**, and SF-36 PCS scores **(F)** among moderate-to-severe plaque psoriasis patients with a BMI <24 kg/m^2^, 24≤ BMI <28 kg/m^2^, and BMI ≥28 kg/m^2^. Comparisons of these variables among the three groups were carried out via ANOVA. *P* < 0.05 indicated statistical significance.

The mean DLQI score from W0 to W52 decreased first but then tended to remain stable in patients with different BMIs. Higher BMI was related to increased mean DLQI scores from W4 to W12 and W36 (Figure 3A). The mean WI-NRS score from W0 to W52 first decreased and then remained stable. The scores from W0 to W52 did not vary among patients with different BMIs (all *P* > 0.05) (Figure 3B). The mean EQ-5D utility index and EQ-5D VAS score from W0 to W52 gradually increased and remained stable. There was no discrepancy in the mean EQ-5D utility index or EQ-5D VAS score from W0 to W52 among patients with different BMIs (all *P* > 0.05) (Figures 3C,D). The SF-36 MCS and SF-36 PCS from W0 to W52 first increased and then remained stable. The SF-36 MCS and SF-36 PCS at most evaluation time points did not change among patients with different BMIs (Figures 3E,F).

### 3.4 Probability of increasing treatment response in moderate-to-severe plaque psoriasis patients with different BMIs

**FIGURE 4:**
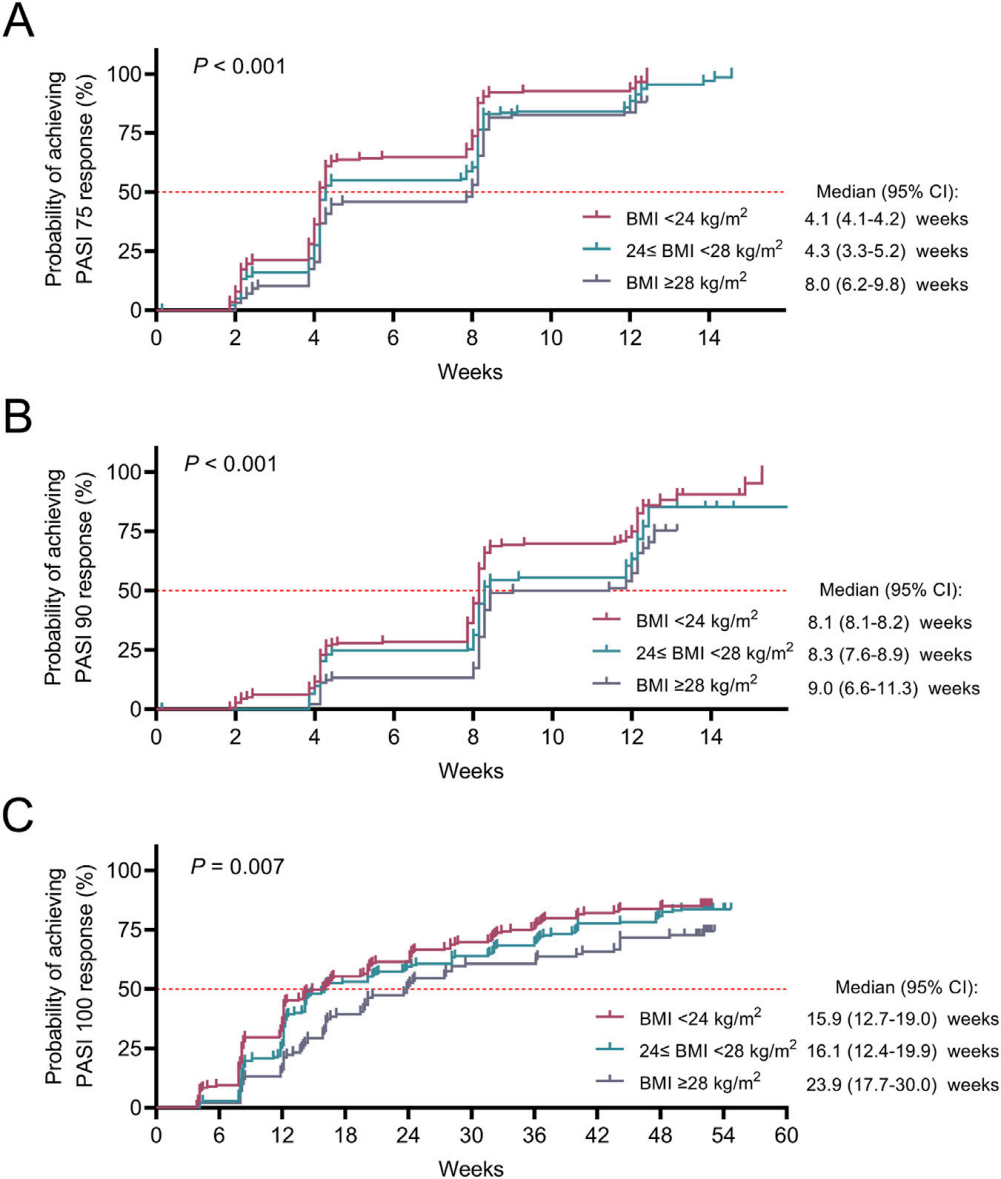
Kaplan‒Meier curve of accumulating treatment response in moderate-to-severe plaque psoriasis patients with different BMIs. Log-rank test for accumulating PASI 75 response **(A)**, PASI 90 response **(B)**, and PASI 100 response **(C)** among moderate-to-severe plaque psoriasis patients with BMI <24 kg/m^2^, 24≤ BMI <28 kg/m^2^, and BMI ≥28 kg/m^2^. The median time to first achieve a PASI score of 75/90/100 among the different groups was compared via the log-rank test. *P* < 0.05 indicated statistical significance.

The probabilities of patients achieving an accumulating PASI 75 response (*P* < 0.001), a PASI 90 response (*P* < 0.001), and a PASI 100 response (*P* = 0.007) were the highest in patients with a BMI <24 kg/m^2^, followed by patients with a 24≤ BMI <28 kg/m^2^, and the lowest in patients with a BMI ≥28 kg/m^2^. The median time was 4.1, 4.3, and 8.0 weeks for achieving a PASI 75 response; 8.1, 8.3, and 9.0 weeks for achieving a PASI 90 response; and 15.9, 16.1, and 23.9 weeks for achieving a PASI 100 response in patients with a BMI <24 kg/m^2^, 24≤ BMI <28 kg/m^2^, and BMI ≥28 kg/m^2^, respectively (Figures 4A–C).

### 3.5 Comparison of the serum concentration of vunakizumab in moderate-to-severe plaque psoriasis patients with different BMIs

**FIGURE 5:**
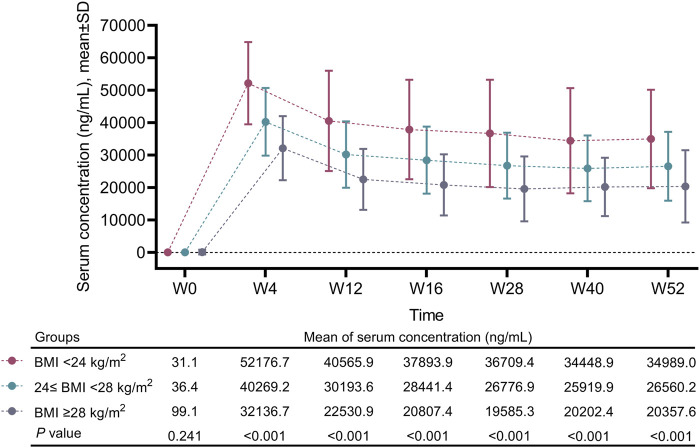
W0--W52 serum concentrations of vunakizumab in moderate-to-severe plaque psoriasis patients with different BMIs. Comparisons of the serum concentrations of vunakizumab among the three groups were carried out via ANOVA.

The mean serum concentration of vunakizumab continuously increased from W0 to W4, then gradually decreased from W4 to W16, and remained stable from W16 to W52 in patients with different BMIs. The mean serum concentration of vunakizumab from W4 to W52 was the highest in patients with a BMI <24 kg/m^2^, moderate in patients with a 24≤ BMI <28 kg/m^2^, and the lowest in patients with a BMI ≥28 kg/m^2^ (all *P* < 0.001) (Figure 5).

### 3.6 Comparison of adverse events in moderate-to-severe plaque psoriasis patients with different BMIs

The incidence of any adverse events was not different among patients with different BMIs, whether in the induction period (*P* = 0.065), maintenance period (*P* = 0.266), or whole period (*P* = 0.494). In terms of specific adverse events, a lower BMI was associated with lower incidences of elevated blood glucose, hyperlipidemia, and hypertriglyceridemia. The incidences of most specific adverse events did not vary among patients with different BMIs (Table 2).

**TABLE 2:** Adverse events.

Events	Induction period				Maintenance period				Whole period			
	BMI <24 kg/m^2^ (n = 179)	24≤ BMI <28 kg/m^2^ (n = 183)	BMI ≥28 kg/m^2^ (n = 99)	*P* value	BMI <24 kg/m^2^ (n = 179)	24≤ BMI <28 kg/m^2^ (n = 183)	BMI ≥28 kg/m^2^ (n = 99)	*P* value	BMI <24 kg/m^2^ (n = 179)	24≤ BMI <28 kg/m^2^ (n = 183)	BMI ≥28 kg/m^2^ (n = 99)	*P* value
Any	64.2%	68.9%	77.8%	0.065	77.7%	77.0%	84.8%	0.266	86.0%	87.4%	90.9%	0.494
Elevated ALT	3.9%	4.9%	9.1%	0.175	8.4%	7.1%	11.1%	0.513	10.6%	9.8%	14.1%	0.530
Elevated blood bilirubin	0.6%	3.8%	3.0%	0.112	2.8%	6.6%	3.0%	0.165	3.4%	8.2%	6.1%	0.145
Elevated blood cholesterol	2.8%	3.8%	3.0%	0.850	2.8%	4.4%	6.1%	0.413	5.0%	6.6%	8.1%	0.593
Elevated blood glucose	1.1%	3.8%	11.1%	<0.001	1.7%	6.6%	13.1%	<0.001	2.8%	8.7%	19.2%	<0.001
Elevated blood TG	6.1%	6.0%	8.1%	0.773	3.4%	2.7%	7.1%	0.177	7.3%	6.6%	11.1%	0.373
Elevated blood UA	3.4%	6.0%	8.1%	0.225	4.5%	7.1%	5.1%	0.532	6.1%	10.4%	9.1%	0.339
Eczema	1.1%	2.2%	2.0%	0.717	4.5%	6.6%	3.0%	0.396	5.0%	7.7%	4.0%	0.390
Elevated AST	1.7%	1.1%	5.1%	0.079	3.9%	2.2%	5.1%	0.418	5.6%	2.7%	9.1%	0.069
Elevated LDL	2.8%	4.4%	1.0%	0.283	3.4%	4.9%	8.1%	0.222	4.5%	6.6%	8.1%	0.453
Hypercholesterolemia	2.2%	3.8%	3.0%	0.678	2.8%	3.3%	6.1%	0.357	3.9%	5.5%	6.1%	0.682
Hyperlipidemia	6.1%	9.8%	7.1%	0.405	4.5%	11.5%	13.1%	0.020	8.4%	16.9%	15.2%	0.046
Hypertriglyceridemia	3.4%	6.0%	14.1%	0.002	3.9%	8.2%	8.1%	0.197	5.0%	12.0%	14.1%	0.021
Hyperuricemia	10.1%	11.5%	9.1%	0.806	10.1%	14.2%	16.2%	0.289	16.8%	19.7%	21.2%	0.621
Injection site reaction	7.3%	4.4%	9.1%	0.269	8.4%	4.9%	4.0%	0.245	12.8%	8.2%	12.1%	0.327
Urticaria	3.4%	4.4%	2.0%	0.585	5.6%	2.2%	3.0%	0.212	7.3%	6.0%	4.0%	0.559
URTI	8.4%	6.0%	12.1%	0.204	19.0%	13.1%	18.2%	0.281	24.6%	17.5%	25.3%	0.175

ALT, alanine aminotransferase; TG, triglyceride; UA, uric acid; AST, aspartate aminotransferase; LDL, low-density lipoprotein; URTI, upper respiratory tract infection.

## 4 Discussion

It is worth noting that in a state of obesity or increased adipose tissue, adipose tissue is not only an energy storage device but also an active endocrine/immune regulator. Upon this backgroud, the efficacy of IL-17A inhibitor (including the vunakizumab) may be weakened. Firstly, adipocytes and their microenvironment (including stromal cells) can secrete a large amount of leptin, which has been proven to promote the differentiation of Th17 cells and enhance the production of IL-17A (Reis et al., 2015). Secondly, adipose tissue is one of the important sources of IL-6 (Popko et al., 2010). IL-6 can drive Th in conjunction with TGF-β through pathways such as STAT3. The differentiation of 17 cells, thereby enhancing the production of IL-17A (Zhang et al., 2021). Under this background, even if IL-17A is neutralized or inhibited, the pro-Th17 signals such as leptin/IL-6 continuously secreted by adipose tissue may maintain a relatively high IL-17A production state, making it difficult for anti-IL-17A treatment to achieve the expected inhibition depth. Furthermore, from the perspective of pharmacokinetics, obese individuals have a larger body weight, a higher proportion of adipose tissue, and changes in blood flow distribution and the volume distribution (Vd) of antibody/protein drugs, accompanied by an increase in clearance or increased volume of distribution. This leads to a decrease in bioeffective exposure per unit dose (i.e., a high body weight/high fat state may be associated with high clearance rate/low antibody exposure) (Gerhart et al., 2022). This means that in an obese state, even if a standard dose is given, the concentration of antibodies in the body that can neutralize IL-17A may be low, thereby weakening the treatment response.

Our study assessed the treatment response to vunakizumab in moderate-to-severe plaque psoriasis patients with different BMIs. A lower BMI was associated with higher W0--W12 accumulating PASI 75, PASI 90, PASI 100, and sPGA 0/1 response rates in moderate-to-severe plaque psoriasis patients who received vunakizumab. Moreover, a lower BMI tended to be related to a greater treatment response from W0 to W52 in these patients. These findings are similar to the results of previous studies, which revealed that a lower BMI was associated with a greater treatment response to biological agents in patients with psoriasis (Huang et al., 2020; Rompoti et al., 2023; Mastorino et al., 2022). This might be because the increase in BMI was associated with increased skin inflammation, elevated leptin, and reduced adiponectin, which was further linked with increased progression of psoriasis, thus leading to a lower treatment response to vunakizumab (Baran et al., 2015; Kaushik et al., 2023; Barr et al., 2022; Guo et al., 2022). There were also two exciting findings in this study. The first one was that the PASI 100 response rates at W36 reached as high as 71.5%, 58.5%, and 50.5% in patients who received vunakizumab with BMI <24 kg/m^2^, 24≤ BMI <28 kg/m^2^, and BMI ≥28 kg/m^2^, respectively, and this effect persisted until W52, with rates of 66.5%, 65.6%, and 52.5%, respectively. These results indicated that vunakizumab had a good effect on skin clearance, even in patients with a BMI ≥28 kg/m^2^. The second difference was that the median time to achieve a PASI 75 response was 4.1 and 4.3 weeks in patients with a BMI <24 kg/m^2^ and 24≤ BMI <28 kg/m^2^, respectively, which indicated that vunakizumab acted rapidly in these patients. Even though the main finding of this study was similar to the previous studies, the magnitude of BMI effect on the efficacy outcome was different.

Only one previous study compared the quality of life of moderate-to-severe plaque psoriasis patients with different BMIs who received a biological agent (infliximab), which revealed that BMI did not affect the quality of life of these patients (Petridis et al., 2018). Our study assessed PROs (including quality of life and degree of itch) in moderate-to-severe plaque psoriasis patients with different BMIs who received vunakizumab. The results revealed that higher BMI was related to increased mean DLQI scores but was not associated with the mean WI-NRS score, EQ-5D utility index, EQ-5D VAS score, SF-36 MCS, or SF-36 PCS. Overall, a lower BMI was associated with a greater quality of life to some extent but was not related to the degree of itch in moderate-to-severe plaque psoriasis patients who received vunakizumab. The inconsistency between our results and those of the previous study might be due to the differences in statistical methods and BMI grouping criteria. The previous study used multiple logistic regression analysis to assess the association of BMI with quality of life, and patients were grouped according to 18.5≤ BMI <25 kg/m^2^, 25≤ BMI <30 kg/m^2^, and BMI ≥30 kg/m^2^ (Petridis et al., 2018).

Previous studies have shown that a lower BMI is associated with increased mean serum concentrations of biological agents (infliximab and secukinumab) in patients with psoriasis (Colls-Gonzalez et al., 2021; Augustin et al., 2022). Similar to the results of these studies (Colls-Gonzalez et al., 2021; Augustin et al., 2022), in our study, a lower BMI was related to a higher mean serum concentration of vunakizumab in moderate-to-severe plaque psoriasis patients. Our findings suggest that moderate-to-severe plaque psoriasis patients with a higher BMI might need more frequent dosing or a higher dose of vunakizumab to obtain greater clinical benefits.

There was no discrepancy in the incidence of any adverse events among moderate-to-severe plaque psoriasis patients who received vunakizumab with different BMIs, whether in the induction period, maintenance period, or whole period. In terms of detailed adverse events, the incidences of the most specific adverse events did not vary among patients with different BMIs. However, BMI was associated with lower incidences of elevated blood glucose, hyperlipidemia, and hypertriglyceridemia. The possible explanations suggested by previous studies were as follows: an increase in BMI was linked with elevated leptin and reduced adiponectin in patients with psoriasis (Baran et al., 2015; Kaushik et al., 2023); moreover, the elevation of leptin and the reduction in adiponectin inhibited insulin secretion, which further increased blood glucose and induced dyslipidemia, thus increasing the incidence of elevated blood glucose, hyperlipidemia, and hypertriglyceridemia (Kirichenko et al., 2022; Jin et al., 2023; Klop et al., 2013). Overall, the above results revealed the favorable safety profile of vunakizumab in moderate-to-severe plaque psoriasis patients, regardless of their BMI.

Notably, our study was a *post hoc* analysis of data from a randomized, controlled phase III trial (NCT04839016) (Yan et al., 2025). Our study divided moderate-to-severe plaque psoriasis patients from the main study into groups according to BMI, and there were some differences in the clinical characteristics among patients with different BMIs, including age, sex, and the proportions of hypertension and hyperuricemia. These differences were possibly caused by the different BMIs of the patients, which might have affected the results of this *post hoc* analysis to some extent.

Several limitations should be noted in our study (Bhagwat and Madke, 2023): The mismatched number of moderate-to-severe plaque psoriasis patients with different BMIs might have interfered with the statistical effect (Mrowietz et al., 2024). All included patients were Chinese, which might limit the generalizability of the results (Sugumaran et al., 2024). Our study was a *post hoc* analysis of a phase III trial (NCT04839016), and future investigations are needed to validate these results (Damiani et al., 2021). Age, sex, hypertension, and hyperuricemia vary among different BMI groups and might also be cofounders that affect treatment efficacy outcomes (Lee and Kim, 2023). Due to the different cut-off value of this study with the previous studies (Crowley et al., 2022; Lin et al., 2025), it was hard to directly compare the magnitude of BMI effect on the efficacy outcome with other IL-17 inhibitors (secukinumab, ixekizumab). Further study could be carried out to explore this issue.

In conclusion, a lower BMI is associated with a better treatment response, greater quality of life, and a lower incidence of specific adverse events in moderate-to-severe plaque psoriasis patients who receive vunakizumab. However, it seems that the efficacy of vunakizumab is relatively poor in the patients with BMI ≥28 kg/m^2^. Therefore, clinicans could consider to increase the dosage of vunakizumab in these patients, such as shortening the dosing interval or increasing the single dose. However, to balance the efficacy and safety outcome, the most suitable dosage should be further explored.

## Data Availability

The original contributions presented in the study are included in the article/Supplementary Material, further inquiries can be directed to the corresponding author.
