# Supplementary material for: Efficacy and safety of vunakizumab in moderate-to-severe plaque psoriasis patients with different body mass index: a post hoc analysis based on a phase III trial
Source: Front Pharmacol. 2026 Jan 6;16:1685072. doi: 10.3389/fphar.2025.1685072 (PMC12817065; doi:10.3389/fphar.2025.1685072)
Supplement: Supplementary file 1 [file Table1.docx]

***Supplementary Materials***

**Supplementary Table 1.** Multivariable logistic regression analysis of BMI and PASI responses.

| Items | *P* value | OR | Lower limit of 95% CI | Upper limit of 95% CI |
| --- | --- | --- | --- | --- |
| **PASI 75 response at W12** |  |  |  |  |
| BMI <24 kg/m^2^ (reference) | (-) | 1.000 | (-) | (-) |
| 24≤ BMI <28 kg/m^2^ vs. reference | 0.212 | 0.517 | 0.183 | 1.458 |
| BMI ≥28 kg/m^2^ vs. reference | 0.004 | 0.222 | 0.080 | 0.614 |
| **PASI 90 response at W12** |  |  |  |  |
| BMI <24 kg/m^2^ (reference) | (-) | 1.000 | (-) | (-) |
| 24≤ BMI <28 kg/m^2^ vs. reference | 0.132 | 0.667 | 0.393 | 1.130 |
| BMI ≥28 kg/m^2^ vs. reference | 0.002 | 0.405 | 0.226 | 0.725 |
| **PASI 100 response at W12** |  |  |  |  |
| BMI <24 kg/m^2^ (reference) | (-) | 1.000 | (-) | (-) |
| 24≤ BMI <28 kg/m^2^ vs. reference | 0.689 | 0.914 | 0.591 | 1.416 |
| BMI ≥28 kg/m^2^ vs. reference | 0.010 | 0.475 | 0.270 | 0.837 |
| **PASI 75 response at W52** |  |  |  |  |
| BMI <24 kg/m^2^ (reference) | (-) | 1.000 | (-) | (-) |
| 24≤ BMI <28 kg/m^2^ vs. reference | 0.376 | 1.344 | 0.698 | 2.585 |
| BMI ≥28 kg/m^2^ vs. reference | 0.914 | 1.042 | 0.498 | 2.181 |
| **PASI 90 response at W52** |  |  |  |  |
| BMI <24 kg/m^2^ (reference) | (-) | 1.000 | (-) | (-) |
| 24≤ BMI <28 kg/m^2^ vs. reference | 0.703 | 0.896 | 0.510 | 1.575 |
| BMI ≥28 kg/m^2^ vs. reference | 0.055 | 0.549 | 0.297 | 1.014 |
| **PASI 100 response at W52** |  |  |  |  |
| BMI <24 kg/m^2^ (reference) | (-) | 1.000 | (-) | (-) |
| 24≤ BMI <28 kg/m^2^ vs. reference | 0.870 | 1.038 | 0.663 | 1.625 |
| BMI ≥28 kg/m^2^ vs. reference | 0.068 | 0.619 | 0.371 | 1.035 |

BMI, body mass index; PASI, Psoriasis Area and Severity Index; OR, odds ratio; CI, confidence interval. The results were adjusted for age, sex, hypertension, and hyperuricemia.

**Supplementary Table 2.** Multivariable logistic regression analysis of PASI responses vs. quartiles of mean concentration.

| Items | *P* value | OR | Lower limit of 95% CI | Upper limit of 95% CI |
| --- | --- | --- | --- | --- |
| **PASI 75 response at W12** |  |  |  |  |
| Q1 (reference) | (-) | 1.000 | (-) | (-) |
| Q2 vs. reference | 0.880 | 1.081 | 0.394 | 2.968 |
| Q3 vs. reference | 0.934 | 1.048 | 0.344 | 3.193 |
| Q4 vs. reference | 0.291 | 2.256 | 0.498 | 10.217 |
| **PASI 90 response at W12** |  |  |  |  |
| Q1 (reference) | (-) | 1.000 | (-) | (-) |
| Q2 vs. reference | 0.592 | 1.184 | 0.639 | 2.193 |
| Q3 vs. reference | 0.324 | 1.399 | 0.718 | 2.728 |
| Q4 vs. reference | 0.146 | 1.766 | 0.821 | 3.798 |
| **PASI 100 response at W12** |  |  |  |  |
| Q1 (reference) | (-) | 1.000 | (-) | (-) |
| Q2 vs. reference | 0.897 | 1.040 | 0.571 | 1.895 |
| Q3 vs. reference | 0.773 | 1.095 | 0.590 | 2.035 |
| Q4 vs. reference | 0.062 | 1.874 | 0.969 | 3.626 |
| **PASI 75 response at W52** |  |  |  |  |
| Q1 (reference) | (-) | 1.000 | (-) | (-) |
| Q2 vs. reference | 0.100 | 1.911 | 0.883 | 4.135 |
| Q3 vs. reference | 0.009 | 3.187 | 1.336 | 7.600 |
| Q4 vs. reference | <0.001 | 8.679 | 2.959 | 25.455 |
| **PASI 90 response at W52** |  |  |  |  |
| Q1 (reference) | (-) | 1.000 | (-) | (-) |
| Q2 vs. reference | 0.002 | 2.814 | 1.454 | 5.446 |
| Q3 vs. reference | <0.001 | 3.382 | 1.653 | 6.917 |
| Q4 vs. reference | <0.001 | 8.114 | 3.300 | 19.949 |
| **PASI 100 response at W52** |  |  |  |  |
| Q1 (reference) | (-) | 1.000 | (-) | (-) |
| Q2 vs. reference | 0.002 | 2.407 | 1.363 | 4.249 |
| Q3 vs. reference | 0.005 | 2.355 | 1.296 | 4.283 |
| Q4 vs. reference | <0.001 | 3.446 | 1.762 | 6.739 |

PASI, Psoriasis Area and Severity Index; OR, odds ratio; CI, confidence interval; Q1, the 1^st^ quartile of mean concentration; Q2, the 2^nd^ quartile of mean concentration; Q3, the 3^rd^ quartile of mean concentration; Q4, the 4^th^ quartile of mean concentration. The results were adjusted for BMI groups, age, sex, smoking, family history of psoriasis (PsO), hypertension, Hyperlipemia, diabetes mellitus (DM), hyperuricemia, and disease duration.
